# Concordance between amyloid PET and CSF biomarkers in clinical setting: a cross-platform comparison and in-depth analysis of discordant cases

**DOI:** 10.1038/s41598-025-23759-5

**Published:** 2025-11-14

**Authors:** Jiří Cerman, Adéla Škorvagová, Martin Vyhnálek, Kateřina Veverová, Kamila Dvořák, Štěpán Kozák, Aleš Kavka, Jakub Hort

**Affiliations:** 1 Department of Neurology, Second Faculty of Medicine, Charles University and Motol University Hospital, Prague, Czech Republic; 2 Department of Natural Sciences, Faculty of Biomedical Engineering, Czech Technical University in Prague, Prague, Czech Republic; 3 PET Centre, Na Homolce Hospital, Prague, Czech Republic

**Keywords:** Alzheimer’s disease, Amyloid positron emission tomography, Cerebrospinal fluid, Centiloid scale, p-tau181/Aβ42 ratio, Biomarker concordance, Biomarkers, Medical research, Neurology, Neuroscience

## Abstract

Reliable detection of amyloid pathology is essential for Alzheimer’s disease (AD) diagnosis and treatment. We directly compared routine ELISA assays and the automated Lumipulse platform against quantitative amyloid PET in a real-world memory clinic cohort. In 153 participants, flutemetamol amyloid PET and CSF biomarkers were assessed across platforms. Concordance with PET and predictors of discordance were evaluated. PET visual reads and Centiloids showed near-perfect agreement (AUC = 0.99). The p-tau181/Aβ42 ratio achieved the highest concordance with PET (OPA 87% ELISA, 92% Lumipulse), while the Lumipulse Aβ42/40 ratio reached 93%. About 6% of participants showed consistent discordance between CSF and PET, associated with APOE ε4 and mixed or non-AD pathologies. Automated CSF assays align strongly with amyloid PET and support biomarker standardization. Persistent discrepancies between CSF and PET likely reflect underlying biological heterogeneity such as mixed or non-AD pathologies and APOE ε4 carriage.

**Supplementary Information:**

The online version contains supplementary material available at 10.1038/s41598-025-23759-5.

## Introduction

The diagnosis and treatment of Alzheimer’s disease (AD) has recently undergone a major transformation with the introduction of the 2024 diagnostic and staging criteria^1^. These build upon the 2018 AT(N) framework^2^, which defines AD as a continuum based on biomarker profiles. The updated guidelines categorize biomarkers into core 1 (A, T1), which are essential for establishing AD pathology and determining eligibility for amyloid-targeting therapies (ATT), and core 2 (T2), which reflect later stages and are currently limited to research use. Core 1 biomarkers include amyloid positron emission tomography (PET), cerebrospinal fluid (CSF) amyloid-β (Aβ), and phosphorylated tau species such as p-tau181 or p-tau217. Core 2 biomarkers include tau PET or emerging fluid markers (e.g., MTBR-tau243)^1^.

The approval of lecanemab and donanemab has further increased the need for biomarker-based diagnosis. Since these treatments require confirmed amyloid pathology—typically by PET or CSF—accurate biomarker classification has become essential^3,4^. Amyloid PET positivity is the most common inclusion criterion in clinical trials because it directly visualizes amyloid deposition, shows high concordance with histopathology^5–7^, and enables monitoring of plaque clearance under the “treat-to-clear” strategy—where ATT can be discontinued once PET confirms substantial plaque clearance^4,8^. PET is usually assessed visually, while semiquantitative staging systems classify cortical and subcortical involvement^9^. Among quantitative methods, the Centiloid (CL) scale is the most widely used and robust^10,11^ harmonizing results and reducing variability in borderline cases.

No universally accepted CL threshold exists: histopathology suggests 12–25 CL indicate moderate-to-high amyloid burden^12,13^, clinical trials use ≥ 36 CL to reduce uncertainty^8,14^, and expert consensus recommends cut-offs between 26–30 CL^15^. In routine care, most scans are read visually, which may be problematic in borderline cases.

CSF biomarkers are a less costly but invasive alternative, widely used in memory clinics. Their main limitation is challenging standardization and inter-laboratory variability^16–18^. Automated platforms such as LUMNIPULSE and Elecsys improve consistency and are increasingly used in research cohorts^19^, but in routine clinical practice manual ELISA assays (e.g., Innotest, Euroimmun) remain far more common in routine practice. Diagnostic interpretation often depends on manufacturer cut-offs, which require local validation^20^. Hybrid ratios such as Aβ42/Aβ40, p-tau181/Aβ42, and t-tau/Aβ42 improve diagnostic accuracy^1^ yet may still yield divergent results^21^.

Overall concordance between PET and CSF is high, but 10–20% of patients show divergent results, more often in clinical than research cohorts^22–25^. Discrepancies may stem from pre-analytical and analytical variability in CSF handling, inconsistent cut-offs^26,27^, borderline or misinterpreted PET findings, and interindividual variation in baseline Aβ levels^28,29^. Biological differences also contribute: PET detects fibrillar amyloid, whereas CSF reflects soluble Aβ42, and the two may diverge in presymptomatic stages. In symptomatic patients, discordance usually indicates heterogeneity due to mixed pathologies or genetic factors such as APOE ε4^30–36^.

Prior studies reported good concordance between automated platforms and ELISA^37–39^ or between CSF assays and PET^37,40–43^, most often using visual reads. To our knowledge, no study has directly performed a cross-platform comparison of ELISA and LUMNIPULSE on the same CSF samples while anchoring classification to quantitative PET. This approach directly addresses key non-biological sources of discordance highlighted above, namely assay variability and uncertainty in PET interpretation.

Given the central role of biomarkers in treatment decisions, this study aimed to:

1. identify the CL threshold with the strongest alignment between visual reads and quantitative PET;
2. compare agreement between CSF and PET across analytes and hybrid ratios, using both conventional ELISA and automated Lumipulse;
3. examine discordant cases in detail, focusing on borderline PET findings and coexisting pathologies.

## Methods

### Cohort characteristics

Participants were recruited from the Czech Brain Aging Study (CBAS)^44^, an ongoing longitudinal, observational, memory clinic-based study. The final study cohort consisted of 153 participants after exclusions (see Supplementary Fig. S4 for details). The patients were referred to the Cognitive Centre, Department of Neurology of the 2nd Faculty of Medicine, Charles University between 2014 and 2024 by general practitioners or other specialists for cognitive complaints reported by themselves or their close persons. All participants provided written informed consent prior to inclusion in the study. All methods were carried out in accordance with relevant guidelines and regulations, including the Declaration of Helsinki and institutional guidelines.

All participants underwent a standard diagnostic workup, which included neurological and laboratory evaluations, a comprehensive neuropsychological examination, *APOE* genotyping, magnetic resonance imaging (MRI; 1.5 or 3 T with MPRAGE sequences), amyloid PET with flutemetamol, and a CSF examination.

Patients were first diagnosed syndromologically and then the clinical etiological diagnosis was determined during a multidisciplinary meeting. The syndromic diagnosis was performed according to the following criteria: mild cognitive impairment (MCI) and dementia were classified in accordance with the National Institute on Aging and Alzheimer’s Association (NIA-AA) recommendations^45–47^, subjective cognitive decline (SCD) was determined in line with the criteria set forth by Jessen^48^. For the etiological diagnosis of AD we used the 2024 AT(N) criteria^1^. The diagnosis of FTLD behavioral variant (bvFTD) was made according to the Rascovski criteria^49^, Gorno-Tempini criteria^50^ were used in cases where the language variant was present and Movement Disorder Society Criteria in diagnosis of progressive supranuclear paralysis (PSP)^51^.

The McKeith criteria were used to diagnose dementia with Lewy bodies (DLB)^52^ and The Diagnostic and Statistical Manual of Mental Disorders, Fifth Edition (DSM-5) for vascular cognitive impairment (VaD)^53^ In cases where a patient meets the criteria for a non-AD dementia condition but also exhibits positive AD biomarkers (e.g., a combination of FTLD + AD or VaD + AD), a diagnosis of mixed pathology was established. Clinical judgment was crucial in determining the predominant contribution of each pathology to the overall clinical presentation in these cases.

### Amyloid PET examination

Amyloid PET was performed on a Biograph 40 TrueV HD PET/CT scanner (Siemens Healthineers) at the Department of Nuclear Medicine and PET Centre, Na Homolce Hospital. A low-dose CT was acquired first for attenuation correction, followed by intravenous injection of [18F]flutemetamol (Vizamyl, GE Healthcare). An early perfusion phase was recorded immediately after injection for 8 min and rebinned into 2-min frames for motion control. The late amyloid phase was acquired 90 min post-injection for a total of 10 min (2 × 5 min). Images were visually rated as positive or negative by two certified nuclear medicine specialists using the GM-EDGE method^54^.

### CL calculation

#### Pipeline validation

The standardized CL pipeline was implemented using the Neurona PET software, based on the SMP8 approach^11^. Structural T1-weighted MRI scans were first preprocessed and aligned to MNI template space (Montreal Neurological Institute). PET images were centered and registered to the MRI scans. Next, unified segmentation of the MRI yielded tissue masks (e.g., c1, c2) and a warp file, which was applied to both MRI and PET images to normalize them into MNI space. This ensured consistent anatomical alignment across modalities.

Region-of-interest (ROI) masks were then applied to extract signal from key regions: cortex, cerebellar grey matter (CG), whole cerebellum (WC), whole cerebellum plus brainstem (WCB), and pons. Standardized uptake values (SUV) and SUV ratios (SUVr) were computed for each ROI.

The pipeline was validated using Pittsburgh compound B (PiB) data, described by Klunk^10^ using the data published on GAAIN platform. Excellent correlation was achieved for all reference regions (R^2^_pons_ = 0,998; R^2^_WC_ = 0,999; R^2^_WC_ = 0,999, R^2^_WCB_ = 0,999), fulfilling the fulfilling validation criteria. Selected as the reference region for further calculations.

SUVr values were then converted to CL using the standard transformation equation^10^.

#### ***Conversion equation [***^***1***^***⁸F]flutemetamol SUVR to CL***

**Fig. 1:**
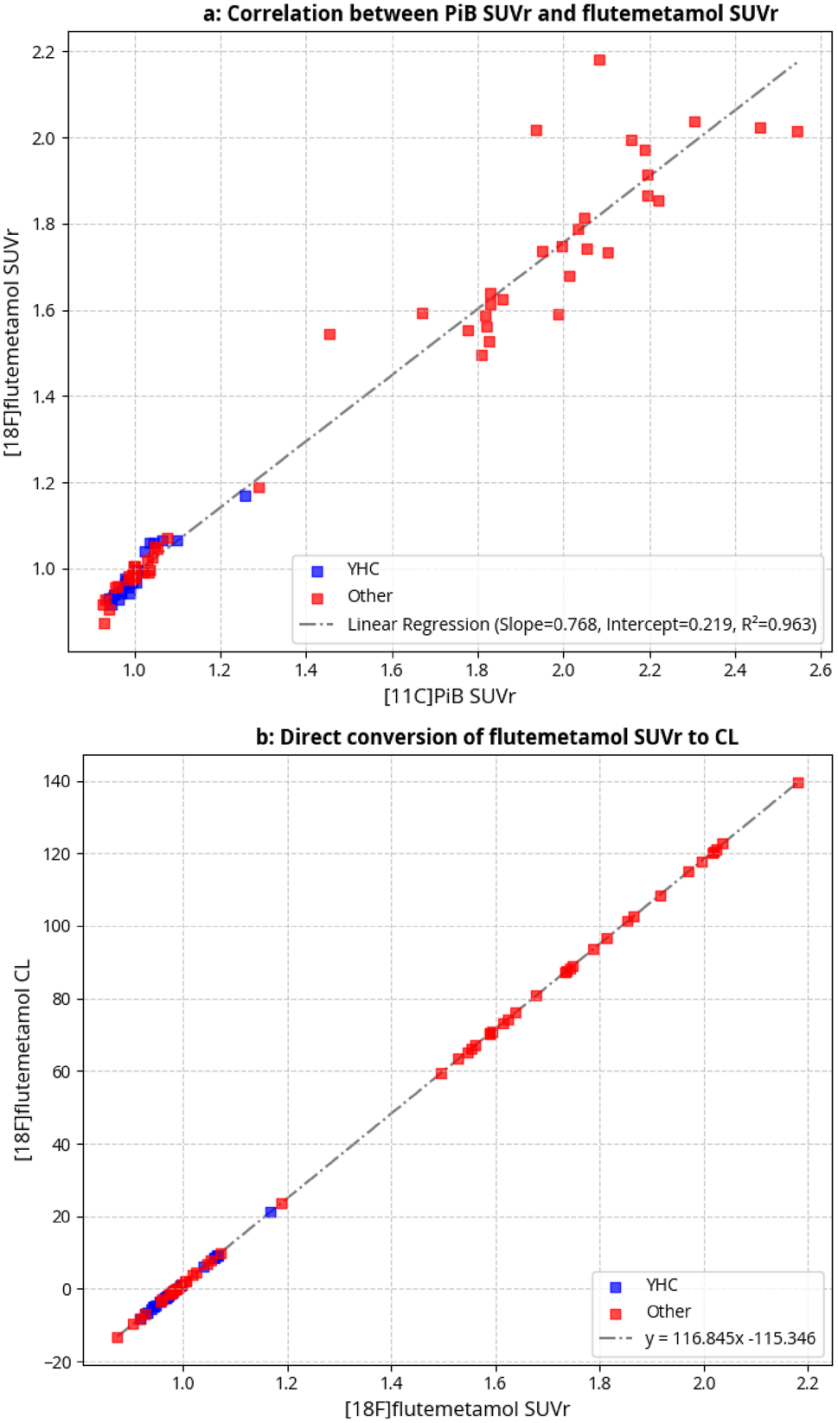
(**a**) Correlation between [^11^C]PiB SUVr and [^1^⁸F]flutemetamol SUVr values processed using the Neurona PET pipeline in 74 subjects from the GAAIN dataset. WC was used as the reference region. Data points labeled “YHC” represent young healthy controls, while “Other” refers to patients with dementia (including both AD and non-AD etiologies). This correlation was used to derive the slope and intercept for PiB-equivalent SUVr transformation. (**b**) Linear equation for direct conversion of flutemetamol SUVr to CL based on the PiB-equivalent SUVr transformation derived from Fig. 1a.

To convert [18F]flutemetamol PET data into CL, paired PiB and flutemetamol scans with corresponding MRI images from 74 subjects (GAAIN dataset) were processed using the same pipeline^11^. a correlation between PiB SUVr and flutemetamol SUVr was calculated (Fig. 1a). A linear transformation was derived by correlating flutemetamol SUVr with PiB SUVr (Fig. 1a). PiB-equivalent SUVr values were then converted to CL using the standard equation: CL = 100 × (PiB-equivalent SUVr − PiB SUVr_YC-0_)/(PiB SUVr_AD-100_ – PiB SUVr_YHC-0_), where YHC-0 corresponds to the GAAIN dataset of young healthy control subjects and AD-100 to the subjects with confirmed AD. The final conversion formula for flutemetamol SUVr to CL is shown in Fig. 1b.

### CSF examination

CSF was obtained by lumbar puncture in the supine position using an atraumatic needle. The first 3 ml were used for routine clinical analyses, and the remaining sample was centrifuged, aliquoted, and stored at − 80 °C within 30 min of collection, following European guidelines^55^. Samples were analyzed at the CSF Laboratory, Institute of Immunology and Department of Neurology, 2nd Faculty of Medicine, Charles University and Motol University Hospital. Until 2019, 92 samples were measured using the Innotest ELISA (Innogenetics, Ghent, Belgium; Aβ42, p-tau181, t-tau). Thereafter, 66 samples were analyzed with the Euroimmun ELISA (Euroimmun, Lübeck, Germany; Aβ42, Aβ40, p-tau181, t-tau) and five samples were tested with both assays. The manufacturer has provided the following cut-offs: Aβ42 > 550 pg/ml, p-tau181 < 61 pg/ml, Aβ42/40 ratio > 0.1 for Euroimmun; Aβ42 > 592 pg/ml, p-tau181 < 71 pg/ml for Innogenetics.

Additionally, 98 archived samples were reanalyzed at the Department of Psychiatry and Neurochemistry, University of Gothenburg, using the automated LUMIPULSE G600II platform (Fujirebio, Ghent, Belgium; Aβ42, Aβ40, p-tau181, t-tau)^56^. The laboratory provided the following cut-offs: Aβ42 > 620 pg/ml, p-tau181 < 61 pg/ml, t-tau < 479 pg/ml, Aβ42/40 ratio > 0.061.

### Statistical analysis

Statistical analyses were performed using IBM SPSS (v26) and R. Descriptive statistics characterized the cohort, and group differences were tested with ANOVA or chi-square.

Receiver Operating Characteristic (ROC) curve analyses were conducted, and the Area Under the Curve (AUC) was calculated to assess diagnostic performance. Based on prior literature, we focused on Centiloid (CL) thresholds of 20 (early amyloid accumulation)^57^, and CL (eligibility for ATT)^15^, reflecting sensitivity versus specificity priorities. CSF biomarkers (Aβ42, p-tau181, p-tau181/Aβ42, Aβ42/40, t-tau/Aβ42) were evaluated against PET at both thresholds. Optimal cut-offs were derived from the Youden J index, and pairwise ROC comparisons were performed using DeLong’s test (pROC package in R) with Holm–Bonferroni correction (see Supplementary Table S1).

Concordance was expressed as overall percent agreement (OPA). Discordant cases were classified as CSF positive and PET negative (CSF + /PET−) or CSF negative and PET positive (CSF−/PET +). Multinomial logistic regression was applied with group status as the dependent variable and APOE genotype, CL value, time interval between PET and lumbar puncture as predictors.

### Study design

This study was specifically designed to address two key non-biological sources of PET–CSF discrepancies: assay variability and uncertainty in PET interpretation. ELISA and Lumipulse assays were performed on aliquots from the same lumbar puncture, collected and processed under identical conditions. This approach eliminated pre-analytical and biological fluctuations as potential confounders. Previous studies often analyzed different cohorts or relied mainly on visual PET^37–39^. Large multicenter initiatives such as BioFINDER and ADNI included quantitative PET, but they did not perform cross-platform comparisons of CSF assays on the same samples.^40^. In contrast, we carried out a within-cohort, side-by-side comparison anchored to Centiloid PET. This design reduces analytical noise and provides stronger evidence for separating true biological discordance from methodological artefacts, thereby extending biomarker concordance research into a real-world clinical setting.

## Results

### Study population

The study included 153 participants: 13 with SCD, 82 with MCI, and 58 with dementia. Participants with SCD were significantly younger than those with MCI (p = 0.002) or dementia (p = 0.027). Groups did not differ in gender or education. As expected, MMSE scores declined progressively across disease stages. Amyloid PET positivity increased from 23% in SCD to 50% in MCI and 62% in dementia (p = 0.033). Mean CL values followed a similar trend but the group difference did not reach significance (p = 0.060). APOE ε4 carrier status was comparable across groups (p = 0.479). The interval between lumbar puncture and PET was longest in the SCD group (vs. MCI: p = 0.017; vs. dementia: p = 0.038). Full demographic data are presented in Table 1.

**Table 1:** Demographic and clinical characteristics of the study participants by diagnostic group.

Characteristic	Overall^1^	SCD^1^	MCI^1^	DEM^1^	p-value
	N = 153	N = 13	N = 82	N = 58	
Sex					0.172
Female	83 (54%)	9 (69%)	39 (48%)	35 (60%)	
Male	70 (46%)	4 (31%)	43 (52%)	23 (40%)	
Education (Y)	14.54 (± 3.19)	15.92 (± 3.17)	14.94 (± 3.10)	13.96 (± 3.16)	0.065
Age at PET (Y)	67.80 (± 9.63)	62.38 (± 8.69)^†^	71.43 (± 9.08)*^†^	67.58 (± 7.76)*	0.001
MMSE	24.98 (± 5.30)	29.08 (± 0.79)^†,‡^	25.43 (± 3.28)^†,^**	18.95 (± 5.41)^‡,^**	< 0.001
Amyloid PET positive	80 (48%)	3 (23%)*^,†^	41 (50%)^†^	36 (62%)*	0.033
Centiloid	38.74 (± 45.61)	16.51 (± 31.65)	38.47 (± 45.90)	48.79 (± 47.08)	0.060
ε4 alle					0.479
Noncarrier	91 (60%)	8 (62%)	51 (62%)	32 (55%)	
Heterozygot	51 (33%)	5 (38%)	27 (33%)	19 (33%)	
Homozygot	11 (7%)	0 (0%)	4 (5%)	7 (12%)	
Days between PET and LP	476.20 (± 504.63)	780.08 (± 513.87)*^,†^	415.62 (± 553.37)^†^	473.34 (± 427.76)*	< 0.001

Values are presented as mean (standard deviation) or counts (percentages), as appropriate. Group comparisons were conducted using ANOVA with subsequent Tukey HSD post hoc test or chi-square test. The threshold for amyloid positivity is > 20 CL.

n (%); Mean (± SD)

*p < 0.05, **p < 0.001, ^†^p < 0.05, ^‡^p < 0.001

The relatively low number of SCD participants reflects clinical barriers in this group: amyloid PET is not reimbursed for cognitively unimpaired individuals in the Czech Republic, and patients with SCD are less willing to undergo lumbar puncture.

### Visual and quantitative PET assessment concordance

Of the 153 flutemetamol PET scans, 77 were visually rated as positive and 76 as negative by two certified nuclear medicine specialists. The mean CL value was 40.5 (SD ± 45.9). Quantitative analysis showed near-perfect agreement with visual assessment (AUC 0.99, 95% CI 0.997–1.001, p < 0.001). The optimal CL threshold was 20.9, yielding 100% sensitivity and 97% specificity (Youden index J = 0.973). A higher threshold of 30.7 CL performed similarly, with 96% sensitivity, 100% specificity, and Youden index J = 0.961.

Visual assessment also identified six patients with cortical positivity but negative striatal uptake. Four of these patients were within the borderline 20–40 CL range and two were above 40 CL (47 and 63 CL), consistent with clinical trial thresholds.

### CSF biomarkers and quantitative PET correlation and concordance

The two ELISA kits (Innogenetics, Euroimmun) and the LUMIPULSE G600II platform were analyzed separately using ROC curves, with amyloid PET as the reference for amyloid positivity. Two analyses were performed: one at > 20 CL (100% sensitivity; best predictor of visual assessment) and the other at > 30 CL (100% specificity; proposed threshold for ATT eligibility). Corresponding CSF cut-offs were derived for both thresholds to assess the impact of PET definition on CSF classification (Table 2).

**Table 2:** Performance and calculated cut-offs of CSF biomarkers for identifying amyloid PET positivity at different CL thresholds.

CL threshold	Platform	Biomarker	AUC**	95% CI	Calculated cut off
20	Euroimmun	Aβ42/40 ratio	0.893	0.811–0.974	≤ 0.08
		Aβ42	0.883	0.794–0.972	≤ 839 pg/ml
		p-tau181	0.839	0.741–0.937	≥ 83.2 pg/ml
		p-tau181/Aβ42 ratio	0.932	0.868–0.996	≥ 0.08
		t-tau/Aβ42 ratio	0.923	0.889–0.996	≥ 0.7
	Innogenetics	Aβ42	0.855	0.771–0.938	≤ 699 pg/ml
		p-tau181	0.739	0.637–0.842	≥ 57.35 pg/ml
		p-tau181/Aβ42 ratio	0.906	0.837–0.975	≥ 0.1
		t-tau/Aβ42 ratio	0.882	0.806–0.958	≥ 0.7
	Lumipulse	Aβ42/40 ratio	0.917	0.847–0.986	≤ 0.069
		Aβ42	0.843	0.753–0.932	≤ 659 pg/ml
		p-tau181	0.794	0.699–0.889	≥ 54.8 pg/ml
		p-tau181/Aβ42 ratio	0.905	0.825–0.985	≥ 0.08
		t-tau/Aβ42 ratio	0.923	0.856–0.991	≥ 0.6
30	Euroimmun	Aβ42/40 ratio	0.893	0.811–0.974	≤ 0.08
		Aβ42	0.883	0.794–0.972	≤ 866 pg/ml
		p-tau181	0.839	0.741–0.937	≥ 83.2 pg/ml
		p-tau181/Aβ42 ratio	0.932	0.868–0.996	≥ 0.08
		t-tau/Aβ42 ratio	0.923	0.889–0.996	≥ 0.7
	Innogenetics	Aβ42	0.855	0.771–0.938	≤ 699 pg/ml
		p-tau181	0.739	0.637–0.842	≥ 56.05 pg/ml
		p-tau181/Aβ42 ratio	0.906	0.837–0.975	≥ 0.1
		t-tau/Aβ42 ratio	0.882	0.806–0.958	≥ 0.7
	Lumipulse	Aβ42/40 ratio	0.917	0.847–0.986	≤ 0.065
		Aβ42	0.843	0.753–0.932	≤ 659 pg/ml
		p-tau181	0.794	0.699–0.889	≥ 54.8 pg/ml
		p-tau181/Aβ42 ratio	0.905	0.825–0.985	≥ 0.08
		t-tau/Aβ42 ratio	0.923	0.856–0.991	≥ 0.6

**p < 0.001.

Table 2 summarizes the area under the ROC curve (AUC), 95% confidence intervals (95% CI), and calculated cut-off values for different CSF biomarkers measured across three analytical platforms (Euroimmun, Innogenetics, and Lumipulse). The cut-offs correspond to the highest Youden index at the specified CL threshold.

#### Pairwise ROC comparison

On the Innogenetics platform, both the p-tau181/Aβ42 and t-tau/Aβ42 ratios significantly outperformed standalone p-tau181 (p < 0.001 and p = 0.015, adjusted).

On Lumipulse, the p-tau181/Aβ42 and t-tau/Aβ42 ratios also showed higher accuracy than p-tau181 (p = 0.018 and p = 0.029, adjusted). The Aβ42/40 ratio additionally improved discriminative performance (p = 0.004, adjusted).

On Euroimmun, the p-tau181/Aβ42 ratio showed a trend toward better performance than p-tau181 (p = 0.010, unadjusted), but this did not remain significant after Holm–Bonferroni correction (p > 0.05, adjusted).

Within Lumipulse, Aβ42/40 and t-tau/Aβ42 showed slightly higher AUCs than Aβ42 alone (p = 0.049 and p = 0.047, unadjusted), but these differences did not remain significant after correction.

**Fig. 2:**
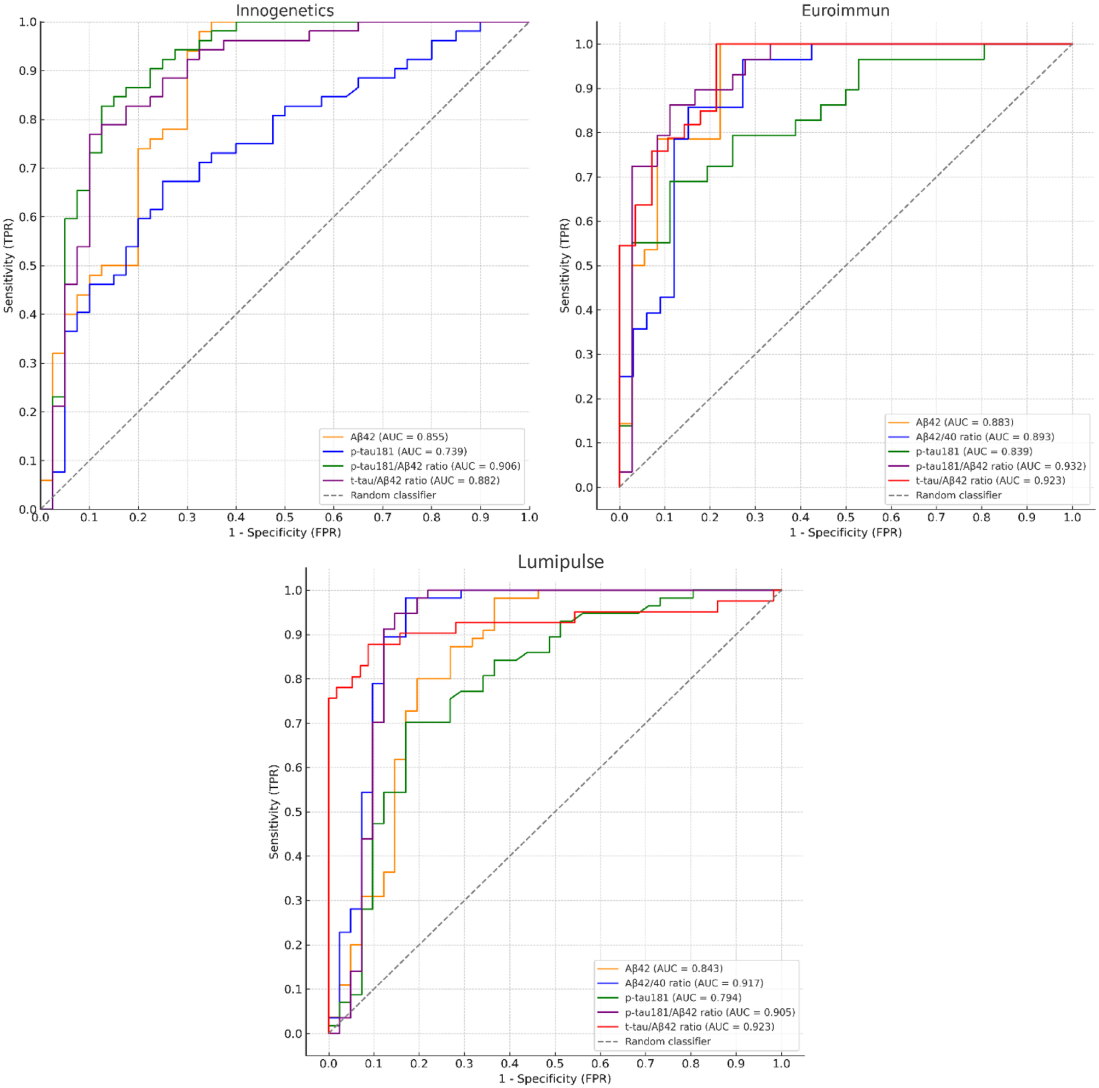
ROC Curves for predicting amyloid PET positivity using CSF biomarkers measured with Innogenetics, Euroimmun, and Lumipulse Assays. ROC curves for cerebrospinal fluid (CSF) biomarkers in predicting amyloid PET positivity, defined as a threshold of > 20 CL, stratified by analytical platform. Each figure displays the performance of different biomarkers or hybrid ratios measured using (**a**) Innogenetics, (**b**) Euroimmun, and (**c**) Lumipulse assays. The plotted biomarkers include Aβ42, Aβ42/40 ratio, p-tau181, p-tau181/Aβ42 ratio, and t-tau/Aβ42 ratio. AUCs are indicated in the legend, all AUCs were statistically significant with p < 0.001.

Overall, the findings align with current recommendations, which discourage using p-tau181 alone due to limited specificity^1,58^. Hybrid ratios generally provided more consistent performance across platforms, although the statistical evidence for superiority may have been limited by sample size. In cross-platform comparisons, Lumipulse showed a consistent trend toward higher AUCs, particularly for p-tau181. The only significant difference was between p-tau181 measured by Innogenetics and Lumipulse (p = 0.025, adjusted). Other trends favoring Lumipulse (e.g., for Aβ42/40 or hybrid ratios) did not reach significance after correction. Full DeLong test results, including unadjusted and adjusted p-values, are provided in Supplementary Table S1 (Fig. 2).

### Overall percentage agreement

Among the evaluated CSF markers, the p-tau181/Aβ42 ratio showed the highest OPA with amyloid PET and was therefore used to illustrate concordance results. At the 20 CL threshold, OPA was 85% for Innogenetics (79 concordant, 14 discordant) and 89% for Euroimmun (59 concordant, 7 discordant). When combined, the two ELISA platforms reached 87% concordance (133 concordant, 20 discordant). Of the five samples measured on both platforms, four were concordant and one discordant. Discordant cases included 5% CSF + /PET− (n = 8) and 8% CSF−/PET + (n = 12).

For the Lumipulse platform (n = 98), OPA for the p-tau181/Aβ42 ratio was higher at 92% (90 concordant, 8 discordant). This included 6% CSF + /PET− (n = 6) and 2% CSF−/PET + (n = 2). Raising the PET threshold to 30 CL did not change OPA for Innogenetics or Euroimmun, which remained 87% combined (133 concordant, 20 discordant). However, two CSF−/PET + cases were reclassified as CSF + /PET−, resulting in a balanced distribution of 6.5% (n = 10) in both discordant categories. Cross-platform agreement between the combined ELISA kits and Lumipulse for the p-tau181/Aβ42 ratio was 87% (85 concordant, 13 discordant).

OPA values for other CSF markers (at the 20 CL threshold) were as follows:

- t-tau/Aβ42 ratio: 82% for Innogenetics (76 concordant, 17 discordant), 89% for Euroimmun (59 concordant, 7 discordant), and 91% for Lumipulse (89 concordant, 9 discordant)
- Aβ42/40 ratio: 84% for Euroimmun (51 concordant, 10 discordant) and 92% for Lumipulse (90 concordant, 8 discordant)
- Aβ42 alone: 82% for Innogenetics (76 concordant, 16 discordant), 83% for Euroimmun (54 concordant, 12 discordant), and 82% for Lumipulse (80 concordant, 18 discordant)

**Fig. 3:**
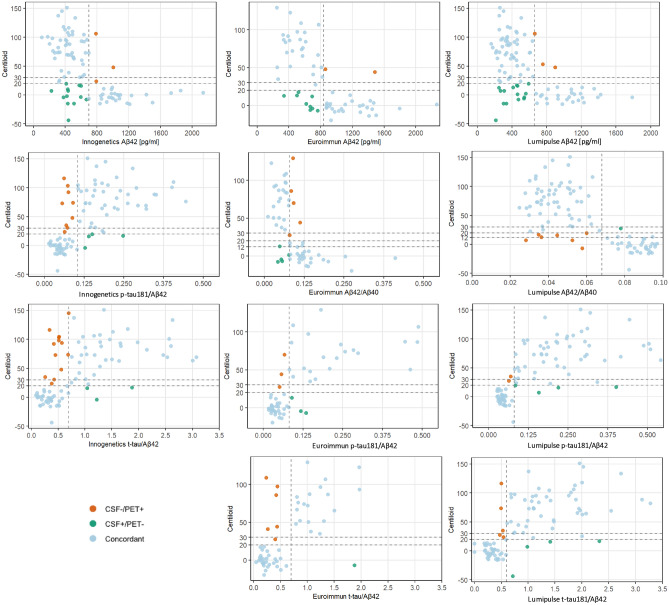
Concordance between amyloid PET status and CSF Biomarkers across Innogenetics, Euroimmun, and Lumipulse platforms. Each graph displays individual subjects plotted according to their CSF biomarker value (x-axis) and corresponding amyloid PET load in CL (y-axis). Concordance is defined by the intersection of a platform-specific CSF cutoff (vertical line) and an amyloid PET threshold of > 20 CL (horizontal line). An additional PET threshold of > 30 CL is also shown, along with a > 12 CL threshold specifically for Aβ42/40 ratio, to illustrate borderline or transitional cases. Concordant positive and concordant negative cases fall in diagonally opposite quadrants; discordant cases (false positives and false negatives) fall outside those regions.

The concordance between amyloid PET and CSF biomarkers across all three assay platforms is summarized in Fig. 3.

Because of an imbalance between CSF + /PET– and CSF–/PET + cases for Aβ42 and Aβ42/40, we tested whether using a lower PET positivity threshold (≥ 12 CL) would improve agreement. With this adjustment, OPA for Aβ42 increased to 85% across all platforms. For the Aβ42/40 ratio, OPA improved to 93% with Lumipulse (91 concordant, 7 discordant), while remaining unchanged at 84% for Euroimmun.

These OPA values for the Lumipulse platform are comparable to those reported in recent multicenter studies. In particular, we replicated the performance of p-tau181/Aβ42 observed in BioFINDER and ADNI^40^, although these cohorts did not include Aβ42/40 measurements or Centiloid anchoring. Similarly, the Mayo Clinic Study of Aging^41^ reported OPA values of 92% for p-tau181/Aβ42 and 91% for Aβ42/40, but without a Centiloid framework. In our cohort, we not only reproduced the established performance of p-tau181/Aβ42 but also demonstrated improved accuracy of Aβ42/40 at a lower Centiloid threshold (12 CL).

### Discrepant cases analysis

Each patient with discordant PET/CSF results was evaluated individually. For this case-by-case analysis, we used the p-tau181/Aβ42 ratio as the primary CSF marker, as it had the highest AUC values and OPA across all platforms.

To verify that discordance was not due to assay variability, Lumipulse analysis was performed on the same CSF sample in 18 of the 20 discordant cases originally identified by ELISA. Seven of these cases remained discordant across both methods. Together with two patients without available Lumipulse results, we defined nine individuals (6% of the cohort) as “true discordant.” This suggests a potential biological rather than technical cause. These included five CSF + /PET− and four CSF−/PET + cases. The small number of true discordant cases (n = 9) limits statistical power, and these results should therefore be interpreted with caution.

In the CSF + /PET− subgroup, four of five patients had Centiloid values above 12 (range 12.4–19.4), and one had a borderline-negative PET (6.9 CL). Three were clinically diagnosed with FTLD (bvFTD or language variant); one of these had autopsy-confirmed mixed AD + FTLD tau pathology. Two other cases met criteria for both AD and vascular dementia (Fazekas score 3 on MRI).

In the CSF−/PET + subgroup, one patient had a clinical diagnosis of PSP (23.5 CL; Euroimmun Aβ42 738 pg/ml, Lumipulse Aβ42 360 pg/ml). Another had a diagnosis of AD (34.9 CL) and a positive Lumipulse Aβ42/40 ratio (0.06), but both striatal amyloid PET and flortaucipir PET were negative, suggesting a very early disease stage. The remaining two patients had high Centiloid values (69.9 and 103.5) with low Aβ42 levels (375 pg/ml and 450 pg/ml), but confirmatory CSF reanalysis was not available. Detailed characteristics of the “true discordant” subgroup are shown in Table 3.

**Table 3:** In-depth biomarker analysis of “true discordant” cases.

Subject	*APOE*	PET			Innogenetics				Euroimmun					Lumipulse					Clinical diagnosis
		Visual read	Stage	CL	Aβ42*	p-tau181*	p-tau/Ab42	t-tau/Ab42	Aβ42*	Aβ42/40	p-tau181*	p-tau/Ab42	t-tau/Ab42	Aβ42*	Aβ42/40	p-tau181*	p-tau/Ab42	t-tau/Ab42	
#1	ε3/ε4	Negative	0	6.9	226	260	1.15	10.15	–	–	–	–	–	311	0.03	275	0.88	5.69	Languague variant FTLD
#2	ε3/ε4	Negative	0	12.4	–	–	–	–	338	0.05	259	0.76	1.74	251	0.04	170	0.68	1.04	bvFTD, autopsy confirmed AD + FTLD-tau
#3	ε3/ε3	Negative	0	15.5	603	84	0.14	1.05	–	–	–	–	–	465	0.04	102	0.22	1.42	Languague variant FTLD, AD comorbidity
#4	ε3/ε4	Negative	0	16.6	578	143	0.25	1.88	–	–	–	–	–	455	0.03	183	0.40	2.33	VaD + AD comorbidity
#5	ε3/ε4	Negative	0	19.4	412	62	0.15	0.67	–	–	–	–	–	593	0.06	50	0.08	0.59	VaD + AD comorbidity
#6	ε3/ε4	Negative	0	27.3	–	–	–	–	738	0.08	38	0.05	0.40	360	0.08	23	0.06	0.48	PSP, AD comorbidity
#7	ε3/ε4	Positive	1	34.9	641	44	0.07	0.26	–	–	–	–	–	501	0.06	35	0.07	0.53	Early MCI due to AD
#8	ε3/ε4	Positive	2	69.9	–	–	–	–	376	0.09	25	0.07	0.65	–	–	–	–	–	MCI due to AD
#9	ε4/ε4	Positive	2	103.5	450	33	0.07	0.52	-	–	–	–	–	–	–	–	–	–	Dementia due to AD

* pg/ml.

In-depth biomarker analysis of “true discordant” cases, defined as individuals whose CSF classification disagrees with amyloid PET positivity (> 20 CL). CSF biomarker status was determined using the p-tau181/Aβ42 ratio, applying platform-specific cutoffs derived to reflect PET positivity above 20 CL. Color coding reflects CSF classification: bold for biomarker-positive, italics for biomarker-negative, and bolditalics for borderline cases. “True discordant” status is defined as the presence of consistent discordance across ELISA-based platforms (Innogenetics or Euroimmun) and Lumipulse, or the absence of a verifying Lumipulse result.

In the final part of the analysis, we examined whether other factors could account for the observed discordant classifications. Two logistic regression models were constructed to predict classification as CSF +/PET–, CSF–/PET + , or concordant. Predictors included APOE ε4 carrier status, Centiloid (CL) value, clinical disease stage (SCD, MCI, dementia), and the time interval between PET and lumbar puncture.

In the first model, which included all discordant cases identified by ELISA, significant predictors were APOE ε4 status (OR = 3.202; p = 0.026) for CSF–/PET + classification, and lower CL values (OR = 0.959; p = 0.026) for CSF + /PET– cases.

In the second model, restricted to the nine “true discordant” cases, a similar but stronger pattern emerged. CSF–/PET + cases were predicted by APOE ε4 status (OR = 8.454; p = 0.030), while CSF +/PET– cases were associated with both APOE ε4 status (OR = 19.3; p = 0.019) and lower CL values (OR = 0.90; p = 0.031). Neither disease stage nor the interval between lumbar puncture and PET were significant in either model. Given the small number of true discordant cases, these regression results should be regarded as exploratory.

## Discussion

In this study, we confirmed high concordance between Centiloid (CL) quantification and visual assessment of amyloid PET. Among CSF biomarkers, the p-tau181/Aβ42 ratio showed the strongest agreement with PET. Analysis of discordant cases revealed that, after excluding assay variability, about half reflected biological heterogeneity related to mixed pathologies, APOE ε4 carriage, or lower amyloid burden. To our knowledge, this is the first study to directly compare ELISA and Lumipulse on the same CSF samples while anchoring classification to quantitative PET.

In the first phase, we validated the CL scale against visual PET reads. A threshold of 20.9 CL yielded 100% sensitivity and 97% specificity. Increasing the threshold to 30.7 CL preserved perfect specificity with only a minor drop in sensitivity, supporting its use as a conservative cut-off for amyloid positivity. Previous studies have reported visual thresholds between 21–26 CL depending on reader expertise^59,60^. By replicating these ranges in a real-world memory clinic cohort, our results extend validation beyond controlled research settings and provide empirical support for applying both 20 CL (sensitive) and 30 CL (specific) thresholds in clinical decision-making^8,13,15,61^.

We identified six individuals with cortical-only positivity and relatively low CL values (median 34). This pattern aligns with borderline cases described in prior studies^9,13,62^ likely reflecting regionally restricted Aβ deposition. Such cases have been associated with slower progression, suggesting a distinct biological subgroup. Their inclusion in ATT trials may dilute treatment efficacy, underscoring the value of quantitative PET for refining eligibility criteria.

Hybrid CSF ratios consistently outperformed single analytes, replicating findings from previous studies. The p-tau181/Aβ42 ratio showed the highest agreement with PET, reaching 87% with ELISA and 92% with Lumipulse, values comparable to single or multicenter reports^37,40–43^. Importantly, by anchoring to Centiloid PET we were able to extend these observations: the Aβ42/40 ratio on the automated platform reached 93% at 12 CL, a histopathological threshold^57^ that also corresponds to donanemab stopping criteria^8^ and is highly relevant for prevention trials^61^. Such analyses were not possible in BioFINDER or ADNI^40^, which lacked Aβ42/40 measures and Centiloid scaling, or in the Mayo Clinic Study of Aging, which measured Aβ42/40 but without a Centiloid anchor^41^. In contrast, Euroimmun ELISA performed robustly at higher thresholds (≥ 30 CL) but less consistently at lower amyloid burden, suggesting that automated platforms may be preferable for early disease stages, while ELISA retains strength in more advanced AD.

A key novelty of our study is the detailed analysis of discordant cases. Previous cohorts such as BioFINDER, ADNI or Mayo Clinic Study of Aging reported discordance rates of ~ 8–10%^40,41^. In our cohort, 20 discrepancies (13%) were initially identified using ELISA, but only 9 (6%) persisted after reanalysis on the automated platform. Thanks to our cross-platform design using aliquots from the same lumbar puncture, we were able to isolate analytical mismatches from true biological heterogeneity. By identifying this “true discordant” subgroup, our study extends multicenter reports^37,40^ and complements earlier single-center studies^41,43^ demonstrating that persistent PET-CSF mismatches are largely driven by mixed or non-AD pathologies and by APOE ε4 carriage, which emerge as major contributors to discordance in symptomatic patients.

The CSF + /PET− subgroup mainly included patients with FTLD or mixed AD/vascular dementia, with one autopsy-confirmed case of mixed AD and FTLD-tau. Most had CL values just above 12, indicating pathology already present but still below the visual detection threshold of PET. In such cases, one would expect only the Aβ42/40 ratio to be abnormal. A positive p-tau181/Aβ42 ratio, in contrast, usually reflects a more advanced stage in which amyloid should already be detectable on PET. Although cerebral amyloid angiopathy (CAA) was considered in two cases, MRI excluded it by modified Boston criteria^63^ and its effect on the p-tau181/Aβ42 ratio appears limited^64–66^.

The PET + /CSF− subgroup comprised four patients. One had PSP with possible AD co-pathology, consistent with reports that tau phosphorylation may be altered in PSP^67^. Another had clinical AD with positive Aβ42/40 but negative striatal and tau PET, fitting an A + T− stage in the NIA-AA framework^1,68^. The remaining two lacked confirmatory automated CSF reanalysis, leaving their classification uncertain. Overall, these patterns show that true discordances are largely attributable to mixed or non-AD pathologies rather than technical error, underlining the value of cross-platform validation.

Our cohort included only 13 SCD patients, reflecting broader clinical realities in the Czech Republic where PET is not reimbursed and lumbar puncture is often declined. Thus, our findings cannot be generalized to preclinical populations. In line with the 2024 NIA-AA criteria^1^, SCD individuals with amyloid biomarkers are classified as stage 2A AD, but their amyloid burden is usually low. The FACEHBI study reported ~ 10% PET positivity and an additional ~ 4% in the “grey zone” (20–35 CL), while subthreshold values (13.5–20 CL) predicted later accumulation in almost 90%^69^. At this early stage, discordance between CSF p-tau181/Aβ42 and amyloid PET is common^70^, and quantitative PET or the CSF Aβ42/40 ratio may provide greater sensitivity for detecting emerging pathology.

Finally, logistic regression identified APOE ε4 carriage and lower CL values as the only significant predictors of discordance, whereas clinical stage and the interval between PET and had no effect. APOE ε4 increased the likelihood of both CSF + /PET− and CSF−/PET + profiles, consistent with its role in impaired amyloid clearance, lipid metabolism, and vascular vulnerability^33^. It is also associated with a higher risk of co-pathologies such as DLB and FTLD^71^. These associations were even stronger in the “true discordant” subgroup, underscoring APOE ε4 as a major biological driver of PET−CSF mismatches.

### Limitations

This study has several limitations. First, histopathological confirmation was not available for most cases. While autopsy could theoretically resolve co-pathologies, it is rarely feasible in clinical cohorts and long delays may allow new changes to emerge. Emerging biomarkers (e.g. α-synuclein seed amplification for DLB) and integrative staging models^1^ may offer better differentiation, though tau PET was not routinely available in our cohort. Second, splitting the sample across two ELISA kits reduced power for cross-platform comparisons, and some DeLong tests lost significance after correction. We therefore report which biomarkers showed the highest agreement with PET rather than claiming statistical superiority. Third, SCD participants were underrepresented, limiting generalization to preclinical AD. Fourth, the Aβ42/40 ratio was not available for all ELISA samples due to a mid-study assay update, which we addressed by stratifying analyses by platform and performing internal concordance checks. Finally, the interval between PET and lumbar puncture varied due to logistical factors, but was not associated with discordance in regression analyses.

## Conclusions

This study demonstrated high concordance between visual and quantitative amyloid PET and strong agreement with CSF hybrid ratios, particularly p-tau181/Aβ42 and Aβ42/40 on the automated Lumipulse platform. By anchoring a cross-platform comparison to quantitative PET, we provide evidence that supports biomarker standardization and highlights the diagnostic utility of automated assays in clinical practice. Importantly, a subset of patients (6%) showed true biological discordance, often linked to APOE ε4 or mixed/non-AD pathologies, underscoring the role of biomarker heterogeneity in symptomatic cohorts. However, the relatively small numbers of SCD participants and “true discordant” cases, together with reduced statistical power in subgroup comparisons due to platform stratification, limit the generalizability of these findings to broader clinical populations.

## Supplementary Information

Supplementary Information.

## Data Availability

Pseudonymized data will be shared by request from a qualified academic investigator for the sole purpose of replicating procedures and results presented in the article and as long as data transfer is in agreement with EU legislation on the general data protection regulation and decisions by Ethics Committee of the University Hospital in Motol.

## Abbreviations

Aβ: Amyloid beta
AD: Alzheimer’s disease
APOE: Apolipoprotein E
AUC: Area under the curve
ATT: Amyloid-targeting therapy
bvFTD: Behavioral variant frontotemporal dementia
CL: Centiloid
CSF: Cerebrospinal fluid
DLB: Dementia with Lewy bodies
ELISA: Enzyme-linked immunosorbent assay
FTLD: Frontotemporal lobar degeneration
LP: Lumbar puncture
MCI: Mild cognitive impairment
MMSE: Mini-Mental State Examination
MRI T1 MPRAGE: Magnetization prepared rapid acquisition gradient echo
OPA: Overall percent agreement
PET: Positron emission tomography
PiB: Pittsburgh compound B
PSP: Progressive supranuclear palsy
ROC: Receiver operating characteristic
ROI: Region of interest
SCD: Subjective cognitive decline
SUVr: Standardized uptake value ratio
VaD: Vascular dementia
WC: Whole cerebellum (used as PET reference region)
