# Supplementary material for: Concordance between amyloid PET and CSF biomarkers in clinical setting: a cross-platform comparison and in-depth analysis of discordant cases
Source: Sci Rep. 2025 Nov 14;15:39963. doi: 10.1038/s41598-025-23759-5 (PMC12618586; doi:10.1038/s41598-025-23759-5)
Supplement: Supplementary file 1 — Supplementary Information. [file 41598_2025_23759_MOESM1_ESM.pdf]

Concordance between amyloid PET and CSF biomarkers in clinical setting: A cross-platform comparison and in-depth analysis of discordant cases

Jiří Cerman[1], Adéla Škorvagová[1], Martin Vyhnálek[1], Kateřina Veverová[1], Kamila Dvořák[2], Štěpán Kozák[3], Aleš Kavka[3], Jakub Hort[1]

Corresponding author: Jiří Cerman,

Email: jiri.cerman@fnmotol.cz

Author details

1) Department of Neurology, Second Faculty of Medicine, Charles University and Motol University Hospital, Prague, Czech Republic
2) Department of Natural Sciences, Faculty of Biomedical Engineering, Czech Technical University in Prague, Czech Republic
3) Na Homolce Hospital, PET Centre, Czech Republic

Supplementary Table S1a: Within-platform DeLong comparison of AUC between CSF biomarkers.

Platform	Comparison	Raw p-value	Adjusted p-value (Holm)
Innogenetics	Aβ42 vs p-tau181	0.126	1
	Aβ42 vs p-tau181/Aβ42	0.076	1
	Aβ42 vs t-tau/Aβ42	0.286	1
	p-tau181 vs p-tau181/Aβ42	0.00002	0.001
	p-tau181 vs t-tau/Aβ42	0.00041	0.015
	p-tau181/Aβ42 vs t-tau/Aβ42	0.086	1
Euroimmun	Aβ42 vs p-tau181	0.6	1
	Aβ42 vs p-tau181/Aβ42	0.08	1
	Aβ42 vs t-tau/Aβ42	0.429	1
	Aβ42 vs Aβ42/40	0.776	1
	p-tau181 vs p-tau181/Aβ42	0.0105	0.347
	p-tau181 vs t-tau/Aβ42	0.518	1
	p-tau181 vs Aβ42/40	0.237	1
	p-tau181/Aβ42 vs t-tau/Aβ42	0.0676	1
	p-tau181/Aβ42 vs Aβ42/40	0.189	1
	t-tau/Aβ42 vs Aβ42/40	0.848	1
Lumipulse	Aβ42 vs p-tau181	0.641	1
	Aβ42 vs p-tau181/Aβ42	0.074	1
	Aβ42 vs t-tau/Aβ42	0.047	1
	Aβ42 vs Aβ42/40	0.049	1
	p-tau181 vs p-tau181/Aβ42	0.00051	0.019
	p-tau181 vs t-tau/Aβ42	0.00086	0.029
	p-tau181 vs Aβ42/40	0.00012	0.004
	p-tau181/Aβ42 vs t-tau/Aβ42	0.535	1
	p-tau181/Aβ42 vs Aβ42/40	0.355	1
	t-tau/Aβ42 vs Aβ42/40	0.721	1

Pairwise comparisons of AUC values among biomarkers measured within the same analytical platform (Innogenetics, Euroimmun, and **Lumipulse**). Comparisons were performed using DeLong's test. Adjusted p-values were calculated using Holm–Bonferroni correction. Corresponding AUC values can be found in Table 2 of the main manuscript.

Supplementary Table S1b: Cross-platform DeLong comparison of AUC for matched biomarker ratios.

Biomarker	Platform comparison	Raw p-value	Adjusted p-value (Holm)
**p-tau181/Aβ-42**	Innogenetics vs Euroimmun	0.47950	1
	Innogenetics vs Lumipulse	0.12282	1
	Euroimmun vs Lumipulse	0.09801	1
**t-tau/Aβ-42**	Innogenetics vs Euroimmun	0.31731	1
	Innogenetics vs Lumipulse	0.04607	1
	Euroimmun vs Lumipulse	0.02981	0.954
**Aβ42/40**	Euroimmun vs Lumipulse	0.29358	1
**p-tau181**	Innogenetics vs Euroimmun	1	1
	Innogenetics vs Lumipulse	0.00072	0.025
	Euroimmun vs Lumipulse	0.14561	1
**Aβ42**	Innogenetics vs Euroimmun	1	1
	Innogenetics vs Lumipulse	0.51440	1
	Euroimmun vs Lumipulse	0.26055	1

Comparison of AUCs for identical biomarkers or their ratios measured across different platforms (Innogenetics, Euroimmun, and Lumipulse). Raw and Holm–Bonferroni adjusted p-values are reported. AUC values for each biomarker or ratio are provided in Table 2 of the main manuscript.

Supplementary Table S2. Mean biomarker concentrations by diagnostic group and assay platform.

Platform	Biomarker	SCD			MCI			Dementia			Total		
		Mean	N	Std. Deviation	Mean	N	Std. Deviation	Mean	N	Std. Deviation	Mean	N	Std. Deviation
Innogenetics	Aβ42 (pg/ml)	726.15	6	449.84	713.04	49	403.28	566.73	37	316.11	655.68	92	376.85
	p-tau181 (pg/ml)	47.00	6	16.94	63.46	49	40.12	79.16	37	44.38	68.64	92	41.63
	t-tau (pg/ml)	275.87	6	102.36	428.68	49	358.45	607.85	37	451.19	490.10	92	399.10
	p-tau181/Aβ-42 ratio	0.09	6	0.06	0.13	49	0.17	0.17	37	0.12	0.15	92	0.14
	t-tau/Aβ-42 ratio	0.50	6	0.32	0.89	49	1.44	1.36	37	1.08	1.06	92	1.28
Euroimmun	Aβ42 (pg/ml)	1014.10	7	312.45	770.23	37	421.15	888.74	22	416.05	836.60	66	412.07
	Aβ40 (pg/ml)	7992.03	7	1683.81	11792.90	37	5664.63	10335.78	22	4006.33	10221.37	66	4880.22
	Aβ42/40 ratio	0.13	7	0.06	0.09	37	0.05	0.11	22	0.08	0.10	66	0.07
	p-tau181 (pg/ml)	41.51	7	24.17	130.35	37	206.84	89.35	22	90.32	106.91	66	164.35
	t-tau (pg/ml)	201.81	7	100.73	567.07	37	547.37	383.38	22	252.93	460.16	66	443.12
	p-tau181/Aβ-42 ratio	0.05	7	0.04	0.23	37	0.36	0.15	22	0.21	0.19	66	0.30
	t-tau/Aβ-42 ratio	0.21	7	0.12	0.95	37	1.04	0.54	22	0.50	0.72	66	0.85
Lumipulse	Aβ42 (pg/ml)	644.60	5	375.64	661.24	55	382.56	442.39	38	218.59	575.53	98	341.87
	Aβ40 (pg/ml)	9945.60	5	2880.40	10173.53	55	3739.18	8976.24	38	3904.54	9697.64	98	3778.41
	Aβ42/40 ratio	0.06	5	0.02	0.06	55	0.02	0.05	38	0.02	0.06	98	0.02
	p-tau181 (pg/ml)	43.06	5	13.41	63.34	55	45.41	96.59	38	75.65	75.20	98	60.42
	t-tau (pg/ml)	282.60	5	85.19	442.51	55	322.13	603.11	38	489.23	496.62	98	397.28
	p-tau181/Aβ-42 ratio	0.09	5	0.07	0.16	55	0.23	0.26	38	0.20	0.20	98	0.22
	t-tau/Aβ-42 ratio	0.58	5	0.36	0.93	55	0.97	1.56	38	1.25	1.16	98	1.11

This table shows mean concentrations, standard deviations, and available sample sizes for each biomarker, stratified by clinical diagnosis (SCD, MCI, and dementia). Aβ42, Aβ40, p-tau181, t-tau are reported in pg/ml. Ratios are unitless. N refers to the number of subjects with available data per biomarker.

Supplementary Table S3: Detailed neuroimaging findings in discordant cases

Subject	Age years	MMSE	PET		MRI									Brain perfusion	Other Biomarkers
			visual	CL	GCA	Faz	MTA	Koed	ERC	PRC	ACC	OFC	ATL		
#1	72	25	negative	6.9	2	2	2/2	2/2	2/2	2.5/2	1.5/1.5	1.5/2	3/2	hypoperfuison left PT	
#2	61	16	negative	12.4	1	1	2/1	2/1	2/2	1/1	1.5/1.5	2/2	2/1	bilat. PT hypoperfusion	
#3	72	15	negative	15.5	2	1	2/1.5	2/2	2/1.5	1.5/2.5	2/2	1/1.5	2/2	na	
#4	69	17	negative	16.6	1.5	3	1.5/1.5	1/1	1/1	2/2	na	na	na	diffuse hypoperf.	
#5	88	30	negative	19.4	2	3	2/2	1/1	1/1	1/1	1/1	0.5/0.5	1/1	normal	
#6	67	21	negative	27.3	1.5	1.5	1.5/1.5	0.5/0.5	1.5/1.5	1/1	0.5/0.5	1.5/1	1/1	diffuse mild hypoperf.	positive DaTSCAN
#7	77	27	positive	34.9	2	1	2/2	0.5/0.5	2.5/2.5	2.5/2.5	1/1	1.5/1	1/1	normal	negative tau PET
#8	76	27	positive	69.9	1	2	2/2	1/1	1/1	1/0.5	0.5/1	1/1	0.5/0.5	diffuse hypoperf.	
#9	67	19	positive	103.5	1	1	3/3	1.5/2	3/3	2.5/2	na	na	na	na	

Overview of visual MRI rating scores in individual patients with discordant amyloid PET and CSF results. Reported scales include global cortical atrophy (GCA)[1], Fazekas score (Faz) for white matter lesions[2], medial temporal atrophy (MTA)[3], Koedam score (Koed) for posterior atrophy[4], and regional atrophy ratings in the entorhinal cortex (ERC), perirhinal cortex (PRC)[5], anterior cingulate cortex (ACC), orbitofrontal cortex (OFC), and anterior temporal lobe (ATL)[6]. Scores are provided separately for the left and right hemispheres where applicable (format: left / right). Visual atrophy scores are based on validated ordinal scales, typically ranging from 0 (no atrophy) to 3 or 4 (severe atrophy), depending on the region. Brain perfusion refers to the early phase of amyloid PET imaging, as described in the Methods section of the manuscript (PT denotes the parietotemporal region).

Supplementary Figure S4: Study population flow

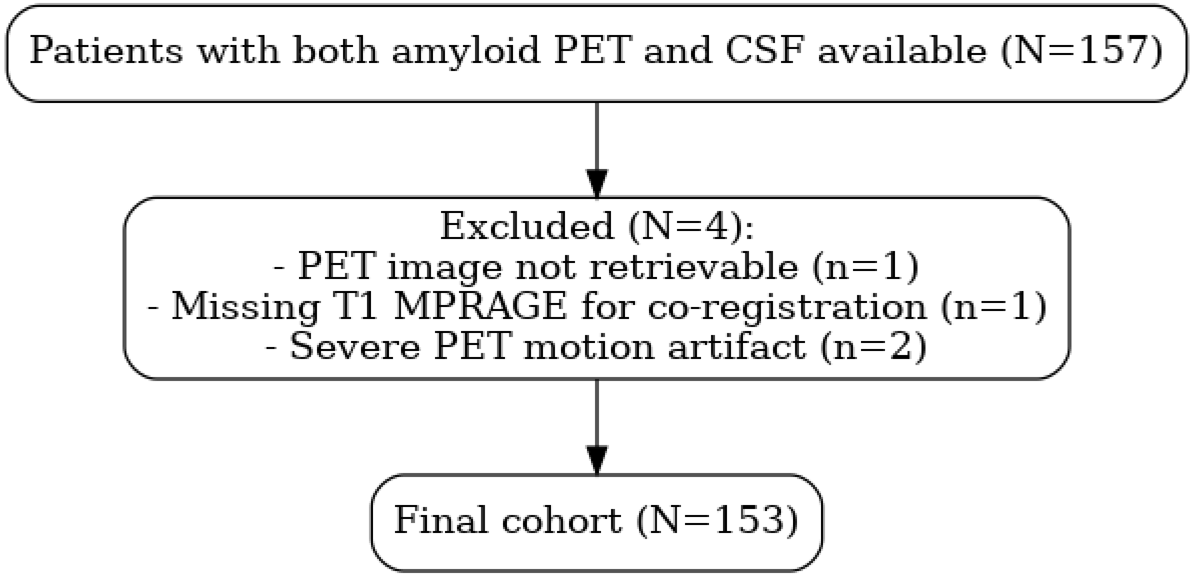

We identified all patients in our memory clinic database who had undergone both amyloid PET and CSF sampling. Four patients were excluded: one due to missing historical PET image, one due to absence of a T1 MPRAGE sequence required for PET/MRI co-registration, and two due to severe PET motion artifacts. The final study sample comprised 153 participants included in the analyses.

References:

1. Pasquier F, Leys D, Weerts JG, Mounier-Vehier F, Barkhof F, Scheltens P. Inter- and intraobserver reproducibility of cerebral atrophy assessment on MRI scans with hemispheric infarcts. Eur Neurol. 1996;36(5):268–72.

2. Fazekas F, Chawluk JB, Alavi A, Hurtig HI, Zimmerman RA. MR signal abnormalities at 1.5 T in Alzheimer's dementia and normal aging. AJR Am J Roentgenol. srpen 1987;149(2):351–6.

3. Scheltens P, Launer LJ, Barkhof F, Weinstein HC, van Gool WA. Visual assessment of medial temporal lobe atrophy on magnetic resonance imaging: interobserver reliability. J Neurol. září 1995;242(9):557–60.

4. Koedam ELGE, Lehmann M, van der Flier WM, Scheltens P, Pijnenburg YAL, Fox N, et al. Visual assessment of posterior atrophy development of a MRI rating scale. Eur Radiol. prosinec 2011;21(12):2618–25.

5. Urs R, Potter E, Barker W, Appel J, Loewenstein DA, Zhao W, et al. Visual Rating System for Assessing Magnetic Resonance Images. J Comput Assist Tomogr. leden 2009;33(1):73–8.

6. Ambikairajah A, Devenney E, Flanagan E, Yew B, Mioshi E, Kiernan MC, et al. A visual MRI atrophy rating scale for the amyotrophic lateral sclerosis-frontotemporal dementia continuum. Amyotroph Lateral Scler Front Degener. 18. červen 2014;15(3–4):226–34.
